# Meningeal Metastasis from Naso-Ethmoidal Malignancies: Pathogenesis, Risk Factors, and Prognostic Impact

**DOI:** 10.3390/jpm15020041

**Published:** 2025-01-22

**Authors:** Remo Accorona, Isabelle Dohin, Davide Mattavelli, Marco Ferrari, Marco Ravanelli, Vittorio Rampinelli, Davide Farina, Piero Nicolai, Cesare Piazza, Alberto Schreiber

**Affiliations:** 1 Unit of Otorhinolaryngology, ASST Grande Ospedale Metropolitano Niguarda, 2062 Milan, Italy; 2 Unit of Otorhinolaryngology—Head and Neck Surgery, ASST Spedali Civili of Brescia, 25123 Brescia, Italyvittorio.rampinelli@asst-spedalicivili.it (V.R.); cesare.piazza@unibs.it (C.P.); alberto.schreiber@asst-spedalicivili.it (A.S.); 3 Department of Medical and Surgical Specialties, Radiological Sciences, and Public Health, University of Brescia, 25123 Brescia, Italy; marco.ravanelli@unibs.it (M.R.); davide.farina@unibs.it (D.F.); 4 Section of Otorhinolaryngology—Head and Neck Surgery, Department of Neurosciences, University of Padova—Azienda Ospedale Università Padova, 35121 Padova, Italy; marco.ferrari@unipd.it (M.F.); piero.nicolai@unipd.it (P.N.); 5 Radiology Unit, ASST Spedali Civili of Brescia, 25123 Brescia, Italy

**Keywords:** meningeal metastases, dural metastases, sinonasal, cancer, recurrence

## Abstract

**Introduction:** Meningeal metastasis (MM) from naso-ethmoidal malignancies (NEMs) is rare, its metastatic route is still debated, and its prognostic impact remains unclear. Our aim is to analyze a retrospective series of NEMs with non-contiguous MM to study the possible route of spread and the prognostic value of MM. **Materials and methods:** The institutional database of SNC treated at the University of Brescia between 1995 and 2021 was reviewed. Clinical–pathological data were collected, and survivals were estimated with Kaplan–Meier. Univariate and multivariate logistic regression analysis were run to identify predictors of MM. **Results:** Among 296 patients, 17 experienced non-contiguous MM, all located along the dura. Intestinal-type adenocarcinoma (10/17) and olfactory neuroblastoma (3/17) were the most frequent histologies. At univariate analysis, brain edema (*p* < 0.0001), resection (*p* = 0.026) or invasion (*p* = 0.006) of brain parenchyma, and local (*p* = 0.0004) and nodal (*p* = 0.021) recurrence were predictors of MM. At multivariate analysis, local recurrence was confirmed as an independent factor (odds ratio: 11.88, *p* = 0.0005). Dural surgical resection was not a risk factor. The five-year disease-specific survival was longer in patients with exclusive MM compared with patients with distant metastasis at other sites (64.3% vs. 30.1% *p* = 0.046). **Conclusions:** Dural venous shunt is the most likely pathway of spread of MM. Local recurrence is the only independent risk factor. Exclusive MM has a better prognosis than extrameningeal metastasis.

## 1. Introduction

The intracranial localization of distant metastases in human cancer is observed in 24% of cases [1,2,3,4]. Brain metastases are the most frequent, while the extra-axial site is less reported, with leptomeningeal metastases being the most represented (4 to 15% of patients with solid tumors), followed by dural metastases (1–9%) and calvarial metastases (1–2%) [1,2,3,4,5,6,7].

Overall, specific tumors show a predilection to brain, meningeal, and calvarial spread. Leptomeningeal metastases are commonly related to breast and ovarian cancer, acute lymphoblastic leukemia, and high-grade non-Hodgkin lymphomas. Metastatic cutaneous melanoma and small cell lung cancer frequently involve both the leptomeninges and brain parenchyma, while non-small cell lung cancer is mostly associated with pure brain metastases. Prostate and kidney cancer are recognized as the main cause of dural and calvarial metastases [2,5,7].

Being an expression of an advanced and systemic disease, meningeal metastasis (MM, including both dural and leptomeningeal spread) has been traditionally considered a poor prognosticator. Indeed, the management of such lesions in the setting of palliative treatment is controversial, including whole-brain radiation therapy (WBRT), radiosurgery, chemotherapy (ChT), and best supportive care (BSC).

MM from head and neck cancers is rare, since only few case reports and small series are published in the literature, and specific mention to MM in naso-ethmoid malignancies (NEMs) is sparse [8,9,10,11,12]. Indeed, the presence of MM from NEM poses different questions, such as the biological nature (distant spread vs. satellitosis/diffusion by contiguity) and the etiology (hematologic/lymphatic spread vs. spread through the cerebrospinal fluid vs. iatrogenic seeding during surgery). As a consequence, it is not obvious that MM from NEM portends the same negative prognostic weight as it is in other cancers, or for metastasis at other sites.

In the present paper, we present a retrospective analysis of patients with MM from NEM treated in a single tertiary referral center, focusing on the possible pathogenesis, pathways of spread, and prognostic value.

## 2. Materials and Methods

The institutional database of patients with NEM treated with curative intent at the Unit of Otorhinolaryngology—Head and Neck Surgery of the University of Brescia between October 1995 and December 2021 was retrospectively reviewed to identify all the cases with distant spread. The following exclusion criteria were used: (a) distant metastasis at presentation, (b) unavailability of preoperative and follow-up MRI for re-evaluation, and (c) unavailability of oncological patient status.

All patients were free of distant metastasis at the time of treatment and developed distant spread during follow-up. Follow-up included periodic clinical evaluations (every 2 months for the first year, every 4 months until the third year, every 6 months until the fifth year, and then annually) and radiological exams (MRI or CT of the head every 4 months for the first 2 years, every 6 months until the fifth year, and then annually; PET-CT or whole-body CT once a year). Examinations were planned irrespective of patients’ symptoms.

Information concerning age, gender, previous treatment, type of surgery, histology, pathological TNM classification, margin status, skull base/dural/brain/olfactory bulb infiltration, brain edema at preoperative magnetic resonance imaging (MRI), lymphovascular invasion (LV), perineural/intraneural spread (PNI), adjuvant treatments, pattern of recurrence, and management strategy of recurrence were retrospectively analyzed. An ancillary analysis of the localization of non-meningeal distant recurrences was performed.

### 2.1. Radiological Analysis

**Figure 1:**
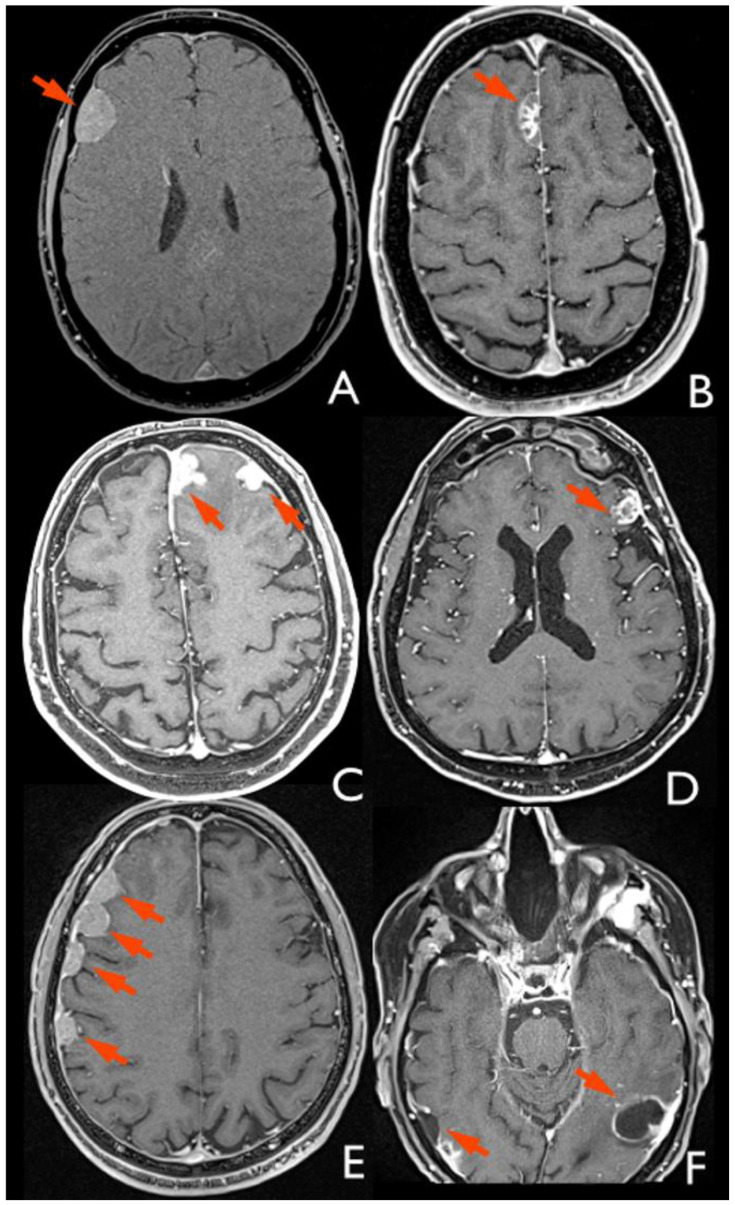
Images of dural implant metastases in MRI on axial plane with VIBE sequences. (**A**) Single metastasis in frontal location; (**B**) single metastasis of the anterior falx; (**C**) dual metastasis in frontal and anterior falx locations; (**D**) single metastasis of the left supraorbital gyrus; (**E**) multiple metastases in fronto-parietal location, some of which are confluent; and (**F**) cystic metastases in right and left occipito-temporal locations.

Preoperative and follow-up MRI studies of patients with MM were retrospectively analyzed by an expert radiologist (M.R.) to define signs of primary tumor transdural extension (i.e., brain edema and skull base/dural/parenchymal infiltration) and classify the recurrent lesions as either leptomeningeal or dural according to the following criteria (Figure 1) [2]:

- Leptomeningeal metastases look like a diffuse coating overlying the parenchyma or multiple small nodules distributed along the cerebrospinal fluid (CSF) spaces.
- Dural metastases show a characteristic “crescent” or biconvex shape, the second usually associated with brain displacement away from the inner table of the skull.

The following MRI sequences were used for the evaluation of MM: TSE-T2, T2-FLAIR, pre- and post-contrast T1 on axial and coronal plane, and post-contrast 3D T1 fat-saturated (VIBE).

In addition, an analysis of the topographical distribution of the lesions was performed. For a better topographical definition, we referred to the cerebral gyri even in the case of dural metastasis (i.e., the MM is at the level of the dura opposite to the corresponding cerebral gyrus).

Isolated recurrences on the inner surface of the dural reconstruction and/or localized within 1 cm from the dural resection were considered local recurrences and therefore were not included in the group of MM.

### 2.2. Statistical Analysis

The association between the collected information and occurrence of MM was verified with a univariate logistic regression analysis. A multivariate logistic regression analysis was run among factors significant at univariate study to identify independent predictors of meningeal metastases. Significance was set at 0.05 and the trend of significance was set between 0.05 and 0.10.

A survival analysis was carried out comparing patients with exclusive MM with patients with extrameningeal metastases with or without meningeal localization. The analysis included the distribution of status at the last follow-up, survival time after primary treatment, latency between primary treatment and distant recurrence, survival after diagnosis of distant recurrence, and Kaplan–Meier analysis after primary diagnosis.

## 3. Results

Among 296 patients included in the institutional database between October 1995 and December 2021, 54 patients (18.2%) met the inclusion criteria and were included in the dataset. The sites of distant spread were as follows: 17 (31.5%) MM, 16 (29.6%) lung metastases, 13 (24.1%) bone metastases, 10 (18.5%) brain metastases, 7 (13.0%) liver metastases, and 10 (18.5%) metastases to other sites (stomach, ileocolic, thigh, uterus, breast, parotid, peritoneum, and mediastinal lymph nodes). In seven (13.0%) patients, the site of distant metastasis was not available. Among patients with MM, nine (52.9%) also showed an extrameningeal localization.

### 3.1. Characteristics of Patients with Meningeal Recurrences

In the group of patients with MM (17 patients), the mean age at the time of surgery was 60 (range: 34–75 years old) and the male-to-female distribution was 13:4. The radiological diagnosis of MM was made by the periodic follow-up protocol and was therefore generally performed before the occurrence of clinical symptoms.

Nine (52.9%) patients had a recurrent tumor at referral to our center. Previous treatments were exclusive surgery in 5 (29.4%), chemotherapy in 3 (17.6%), and surgery and chemoradiation in 1 (5.9%) patient.

All patients underwent surgery with curative intent. Endoscopic resection without anterior skull base craniectomy (ER) was performed in 2 (11.8%) patients, with transnasal craniectomy (ERTC) in 11 (64.7%) cases, and a combined cranioendoscopic approach (CER) in 4 (23.5%) cases. Surgery was extended to brain parenchyma resection in eight cases (47.1%). Demographics and the clinical and radiological features of NEM with MM are described in Table 1.

At the histopathological examination (Table 2), the resection was in uninvolved margins in 11 patients (64.7%), while 6 patients showed microscopic positive margins (35.3%). The most frequent histologic type was intestinal-type adenocarcinoma (ITAC, 10 cases, 58.8%) followed by olfactory neuroblastoma (ONB, 3 cases, 17.6%). Most patients had pT4-class tumors, and all were cN0 at the time of primary treatment. According to the final pathological examination, the tumor invaded the bone of the skull base in 9 (52.9%) patients, the dura in 8 (47.1%) patients, the brain in 6 (35.3%), and the olfactory bulb in 1 case (5.8%). Microscopic LV was observed in 12 (70.6%) cases, while perineural/intraneural spread was observed in 7 (41.2%) cases.

Twelve (70.6%) patients received adjuvant treatments on the primary tumor (Table 1), while in the remainders (5, 29.4%), it was contraindicated because of previous irradiation (3, 17.6%), early pT stage (1, 5.9%), and early post-surgical progression of the disease (1, 5.9%).

Eleven patients (64.7%) showed a local recurrence, of whom 2/17 (11.8%) had a local and distant recurrence and 3/17 (17.6%) had a local, nodal, and distant extra-meningeal recurrence. Four patients presented nodal recurrence (23.5%), in all cases together with local or distant relapse. Distant metastases were reported in nine cases (52.9%). Two patients (11.8%) did not show other recurrences apart from MM. The description of the local, regional, and distant recurrences is detailed in Table 3.

**Figure 2:**
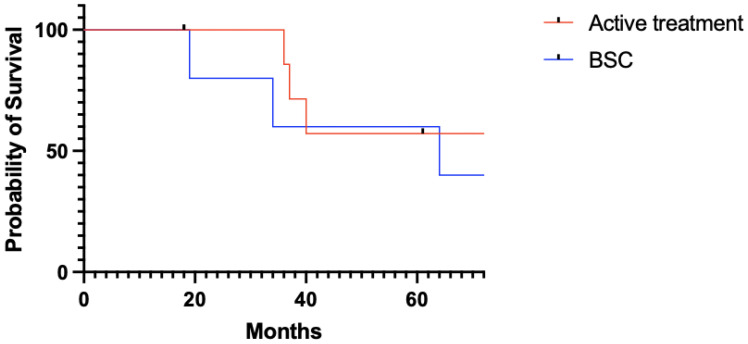
Kaplan–Meier estimates showing overall survival in patients who underwent active treatment versus best supportive care for MM. Active treatment versus best supportive care: median survival, 50.5 months and 49 months, respectively; *p* = 0.344 (log rank test).

Overall, MM was treated in eight (47.1%) patients. Radiotherapy was the most frequent treatment modality: 4 (23.5%) patients underwent WBRT (30 Gy with 3 Gy fractions), while gamma-knife radiosurgery and adrotherapy were delivered to 1 patient each (5.9%). Two patients received systemic therapy, ChT with temozolomide (75 mg/m^2^) and an off-label protocol of lutetium-177-based radiometabolic therapy. BSC was scheduled for the remaining nine (52.9%) patients. Figure 2 shows the overall survival in patients with MM who underwent active treatment versus best supportive care.

### 3.2. Radiological Analysis

The MRI of 15/17 (88.2%) patients with MM was evaluated. The remaining two patients were excluded because the available imaging was of inadequate quality for the purpose of this study.

All the metastases were dural; no leptomeningeal spread was identified. Fourteen (93.3%) patients showed one or more localizations to the dura of the cranial vault, while only one (6.7%) patient presented a single lesion located at the dura of the skull base, more than 1 cm away from the dural reconstruction. Among the 14 patients with cranial vault dural metastases, 4/15 (26.7%) had concomitant lesions at the dura of the skull base. The cranial vault lesions were prevalent in frontal, temporal, and parietal areas. Dural metastases of the skull base were in the anterior and middle cranial fossae. Finally, five (33.3%) patients presented a concomitant localization to the falx cerebri. No relation with the site (left or right) of the primitive tumor was observed. A total of 6 patients had bilateral MM (40%), 5/15 patients presented ipsilateral MM (33.3%), and 4/15 patients showed contralateral MM (26.7%). The topographic distribution of the MM is detailed in Table 3.

### 3.3. Risk Factors for MM and Survival Analysis

An analysis of the association between clinical and pathological factors and the onset of MM is reported in Table 3. At univariate analysis, the presence of brain edema at preoperative imaging (*p* < 0.0001), resection of the brain parenchyma (*p* = 0.026), invasion of the brain parenchyma (*p* = 0.006), local recurrence (*p* = 0.0004), and nodal recurrence (*p* = 0.021) were significantly associated with the occurrence of meningeal metastases. Previous treatment (*p* = 0.093), especially when including chemotherapy (*p* = 0.070), positive surgical margins (*p* = 0.061), dural invasion (*p* = 0.092), and distant extrameningeal metastases (*p* = 0.058), showed a trend of significance.

At multivariate analysis, local recurrence was the only independent predictor of meningeal metastasis (odds-ratio: 11.88, *p* = 0.0005).

The median follow-up was 37 months (range: 11–206 months). At the latest follow-up evaluation, 8 patients (47.1%) were dead of disease, 1 patient was dead of other causes (5.9%), and 8 patients were alive with disease (47.1%). The latency between diagnosis and meningeal recurrence was 31.6 months (range: 1.6–122.9 months). The median survival after the treatment performed at our institution was 35 months (range: 6–178 months). The median survival after the diagnosis of MM was 21 months (range: 10–27 months) in the entire group and 11 months (range: 0–111 months) in the group of patients dead of disease.

**Figure 3:**
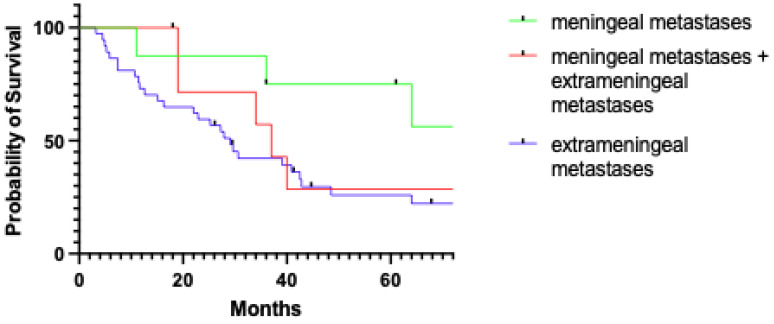
Comparison of disease-specific survival (DSS) between patients with meningeal metastases, patients with meningeal and extrameningeal metastases, and patients with exclusive extrameningeal metastases (log rank *p* = 0.05).

The five-year disease-specific survival (DSS) after the treatment performed at our institution was 64.3% in patients with exclusive MM and 30.1% for patients with extra-meningeal metastases (*p* = 0.035, Figure 3).

## 4. Discussion

To our knowledge, the present single-center study is the largest one focusing on MM from NEM reported in the literature. Our analysis provided some relevant insights. First, the dura was the most frequent metastatic site among the patients with distant relapse. Second, local recurrence of the primary tumor was the only independent risk factor of MM. Third, MM alone portended a better prognosis than extrameningeal metastasis. Finally, we investigated the pathogenesis of MM and identified venous spread as the most likely route of dissemination.

### 4.1. Pathogenesis and Risk Factors

From cancer of any site, the hematogenous route is acknowledged as the primary path of neoplastic spread for MM [2,3,4,5,6,7]. In NEM, the pathogenesis remains controversial and still debated due to the proximity of these tumors to the skull base, which poses a risk of either abutting or infiltrating the meninges. Moreover, the surgical treatment of the tumor may encompass dural resection of the anterior cranial fossa. Indeed, several authors have questioned the classic hematogenous diffusion, advocating iatrogenic CSF seeding as a potential mechanism [2].

The main theories concerning the path of neoplastic spread to the meninges are hematogenous spread, CSF seeding, and lymphatic diffusion. We herein pass through these hypotheses; based on our findings, we support the venous hematogenic pathway.

### 4.2. Hematogenic Spread

The middle meningeal artery system has been suggested as the main route of hematogenous arterial spread to the meninges. Jiang et al. [10] reported a series of 10 patients treated with craniofacial resection and adjuvant radiation therapy for ONB; all cases developed a dural metastasis in the perisylvian region (9 unilateral, 1 bilateral). As this region is the most vascularized within the dural system, the authors suggested that ONB neoplastic emboli should have reached the perisylvian area through the middle meningeal artery system. On one hand, our results partially support this finding, since all the MMs in our patients are located within the dura mater, which exhibits greater vascularity compared to the leptomeninges, and most of the metastases were found in the fronto-temporo-parietal region, which is primarily supplied by the middle meningeal artery system. On the other hand, the significant prevalence of dural metastases over other organs that acts as a filter (i.e., lung, bones, and liver) is against the hematogenous arterial hypothesis. Furthermore, the validity of arterial spread is brought into question by the absence of spinal meningeal metastases in the current series, despite the expectation that a random arterial embolism would also impact the spinal dura. However, if the meninges are considered a venous filter of the naso-ethmoidal region similarly to the liver for colorectal cancer, the high prevalence of dural metastases advocate for a venous spread of the metastases. Indeed, the liver is the first location of metastatic disease from colorectal cancer as the main mechanism of dissemination is through the portal system which directly connects the colorectal cancer and liver, which is associated with abundant blood supply. In view of the similarity between venous and arterial middle meningeal systems [13], the topographical distribution of MM observed in the present and other studies [10] could also advocate for the hematogenous venous shunt hypothesis. Different authors have proposed the venous pathway as an ancillary route for metastatic cells to reach the dura via diploic vessels [2,5,7,14]. Indeed, when considering NEM, their close proximity to the dura may favor the venous shunt and embolization. The dural venous system serves as a crossroad for blood drainage from both the calvarium and the brain. Its intricate and plexiform structure makes it particularly susceptible to embolization, resembling a regional rather than systemic metastatic process [13]. Neoplastic emboli may infiltrate this complex system either directly or through iatrogenic contamination, metastasizing to areas with slow blood flow. Hence, the hematogenous venous shunt hypothesis could explain both the topographical distribution and the dural prevalence over other distant sites.

### 4.3. CSF Seeding

One of the theories advanced to explain MM from NEM concerns CSF seeding (spontaneous or iatrogenic). This metastatic route is well known for several central nervous system (CNS) malignancies, such as medulloblastoma, ependymoma, and pineoblastoma [15]. The idea of spontaneous CSF seeding is supported by the presence of vital neoplastic cells in the CSF of these patients [4,16,17,18,19,20,21]. Moreover, Wen et al. [22] were the first to propose the iatrogenic contamination of the dural space. They described a case of leptomeningeal carcinomatosis from an ethmoid adenocarcinoma treated with craniofacial resection and radiotherapy, suggesting that a breach in the dura during surgery might allow neoplastic cells to directly enter the CSF.

However, our findings do not corroborate this pathway of spread. Dural opening during surgery was not predictive of meningeal metastases, and in two patients, the dura was left intact, precluding the possibility of an iatrogenic CSF seeding. Moreover, studies on CSF physiology highlighted that CSF circulation tends towards major cerebral cisterns, due to both flow dynamics and gravitational fall [23]. As a result, the cerebellomedullary cistern and the lumbar cistern should be the main site of metastases. Conversely, the topographical distribution of MM contradicts the expected pattern in the case of CSF dissemination, as previously noted by Jiang et al. [10].

### 4.4. Lymphatic Diffusion

Lymphatic diffusion has never been mentioned as a possible pathway for MM. Pollay underlined that, in addition to arachnoid granulations, lymphatic drainage serves as a route for CSF outflow [23]. Moreover, several experimental studies have shown that a considerable proportion of CSF is reabsorbed via the lymphatic vessels placed in the perineural sheath of some cranial nerves (i.e., the olfactory nerve) and drained to the cervical lymphatic system [24,25,26]. In this context, retrograde lymphatic spread could theoretically lead to the migration of metastatic cells from the naso-ethmoid complex to the intracranial space. However, there are several arguments against this hypothesis. First, studies on lymphatic CSF outflow are preclinical, and the precise microscopic configuration of the interface between the perineural space and the lymphatic vessel wall remains unknown. Moreover, only a minority of cases included in our series showed PNI and LV.

Finally, based on the hypotheses discussed above and evaluated through deductive reasoning, we propose the venous hematogenic route as the most plausible pathway, acknowledging this as a speculative interpretation.

### 4.5. Prognostic Impact and Principles of Palliative Care

Local recurrence emerged as the sole independent predictor of MM, which represents a hallmark of disseminated disease. However, it is noteworthy that in our study, patients with MM alone had a more favorable outcome and showed prolonged survival compared with patients exhibiting metastasis at other distant sites. Indeed, the disease-specific survival estimates at 5 years were 64.3% and 30.1%, respectively. Concerning patients dead of disease in the two groups, the median time from the diagnosis of metastasis to death was 11 months and 5 months, respectively. These data are in contrast with the mean survival of patients with meningeal metastasis from other solid tumors, which ranges from 3 to 6 months [27,28,29,30]. This difference in survival further supports the concept of MM in NEM as a regional rather than distant spread.

The palliative treatment of MM is not supported by shared guidelines. There are several studies published in the literature on the role of WBRT, intrathecal chemotherapy, and systemic chemotherapy in the palliation for intracranial metastases. However, as supported by our findings, no benefit in terms of survival or quality of life has been demonstrated so far [27,28,31,32,33,34,35,36,37,38,39,40,41,42].

The indication to palliative treatments is on a case-by-case basis, depending on the performance status of the patient, histology, and therapeutic opportunities. The lack of data in the literature, the low number of patients of our series, the variability of recurrence pattern, and the heterogeneity of treatment protocols preclude firm conclusions on the management of MM from NEM. However, the improved oncological outcomes compared with patients with metastatic spread and the reasonable expectation of obtaining prolonged survival may prompt the physicians to consider more favorably the treatment of MM in selected, fit patients.

## 5. Conclusions

In our study, dural metastases represent the most frequent non-contiguous recurrence of NEM. The pathogenesis of MM is controversial; our discussion supports the hematogenous theory via the venous pathway. Patients with MM have a poor prognosis. However, MM alone was associated with a better prognosis and prolonged survival compared with extrameningeal metastasis, thus encouraging the favorable consideration of palliative treatments in selected, fit patients. Further studies are warranted to investigate the best treatment protocols.

## Figures and Tables

**Table 1:** Clinical and demographic characteristics of patients with meningeal metastases.

			*n*	*%*
Gender	Male		13	76.47%
	Female		4	23.53%
Previous treatment	None		8	47.06%
	Surgery		5	29.41%
	ChT		3	17.65%
	Surgery + RT + ChT		1	5.88%
MRI critical relations	Skull base invasion	Yes	9	52.94%
		No	8	47.06%
	Dural invasion	Yes	8	47.06%
		No	9	52.94%
	Brain edema	Yes	6	35.29%
		No	6	35.29%
		NA	5	29.41%
	Brain parenchyma invasion	Yes	5	29.41%
		No	12	70.59%
Surgery	ER		2	11.76%
	ERTC		11	64.71%
	CER		4	23.53%
Brain resection	Yes		8	47.06%
	No		9	52.94%
Adjuvant treatment	RT		9	52.94%
	ChT		1	5.88%
	Chemoradiation		2	11.76%
	None		5	29.41%
Follow-up status	NED		0	0.00%
	AWD		8	47.06%
	DOD		8	47.06%
	DOC		1	5.88%

MRI: magnetic resonance imaging, ChT: chemotherapy, RT: radiation therapy, ER: endoscopic resection, ERTC: endoscopic resection with transnasal craniectomy, CER: cranioendoscopic resection, NED: no evidence of disease, AWD: alive with disease, DOD: dead of disease, DOC: dead of other causes.

**Table 2:** Pathological characteristics of primary tumor in patients with meningeal metastases.

		*n*	*%*
Histology	ITAC	10	58.8%
	ONB	3	17.60%
	SNUC	1	5.9%
	ES	1	5.9%
	CaNOS	2	11.80%
pT	T1	1	5.88%
	T2	2	11.76%
	T3	3	17.65%
	T4a	4	23.53%
	T4b	7	41.18%
Surgical margins	R0	11	64.71%
	R1	6	35.29%
Grade	G1	2	11.76%
	G2	5	29.41%
	G3	10	58.82%
Lymphovascular invasion	Lv0	5	29.41%
	Lv1	12	70.59%
Perineural invasion	Pn0	10	58.82%
	Pn1	7	41.18%
Dural invasion	Yes	8	47.06%
	No	9	52.94%
Skull base invasion	Yes	9	52.94%
	No	8	47.06%
Brain parenchyma invasion	Yes	6	35.29%
	No	7	41.18%
	NA	4	23.53%
Olfactory bulb infiltration	Yes	1	5.88%
	No	16	94.12%

ITAC: intestinal-type adenocarcinomas, ONB: olfactory neuroblastoma, SNUC: sinonasal undifferentiated carcinoma, ES: Ewing sarcoma, CaNOS: carcinomas not otherwise specified.

**Table 3:** Description of clinical and pathological characteristics, pattern of recurrences, and topographic distribution of the patients with meningeal metastases.

	Clinical and Demographic Characteristics					Pathological Characteristics								Recurrences			Meningeal Metastases	
	Gender	Previous Treatment	Surgery	Adjuvant Treatment	FU Status	Histology	Grade	pT	Surgical Margins	Dural Invasion	Skull Base Invasion	Brain Parenchyma Invasion	Olfactory Bulb Invasion	Local	Nodal	Distant (Extrameningeal)	Number	Topographic Distribution
1	M	None	ER	RT	AWD	ITAC	2	2	R1	NA	0	0	0	NE	0	0	NA	NA
2	M	None	ERTC	None	DOD	ITAC	2	2	R0	0	0	0	0	Frontal	0	0	NA	NA
3	M	CHT-RT	CER	CHT	DOC	ES	3	4a	R0	NA	0	0	0	NE + skull base + zygomatic and temporal bone	0	0	3	2 frontal gyrus and 1 ACF/MCF
4	M	CHT	CER	RT	AWD	ITAC	2	4a	R0	NA	0	0	0	Skull base	0	0	1	ACF/MCF
5	M	CHT	CER	RT	AWD	ONB	1	3	R1	1	0	0	0	Maxilla	Cervical	Bone	4	1 frontal gyrus,
																		2 temporal gyrus, 1 ACF/MCF
6	M	None	ER	None	DOD	ITAC	3	1	R0	NA	0	0	0	Maxilla	0	0	1	Temporal gyrus
7	M	None	ERTC	RT	DOD	ITAC	1	4b	R1	0	1	1	0	0	0	Brain	3	Frontal gyrus
8	M	CHT	ERTC	RT	AWD	SNUC	3	4b	R0	1	1	1	0	Skull base	Cervical	Bone	2	1 frontal gyrus, 1 ACF/MCF
																		1 supramarginal gyrus,
9	M	Surgery	ERTC	RT	DOD	ITAC	3	3	R1	0	1	0	0	0	0	Lung	6	5 frontal gyrus, 1 falx cerebri
																		1 falx cerebri, 2 ACF/MCF
10	M	Surgery	ERTC	None	DOD	ITAC	2	3	R0	0	0	0	0	NE + orbit	0	Bone	14	4 frontal gyrus, 4 temporal gyrus,
																		2 postcentral gyrus
11	M	Surgery	ERTC	None	DOD	ITAC	3	4b	R1	1	1	1	0	NE	0	Liver	1	Temporal gyrus
12	W	None	ERTC	RT-CHT	AWD	ONB	3	4b	R1	1	1	1	0	0	0	0	2	1 frontal gyrus, 1 temporal gyrus
13	M	None	ERTC	RT	DOD	ITAC	3	4a	R0	0	0	0	0	Orbit	Cervical	Parotid + thigh	1	Frontal gyrus
14	W	None	ERTC	RT-CHT	AWD	CaNOS	3	4b	R0	1	1	1	0	0	Cervical	Mediastinum	7	1 sylvian fissure, 2 falx cerebri
																		3 frontal gyrus, 1 temporal gyrus
15	M	None	CER	None	DOD	CaNOS	3	4b	R0	1	1	1	0	NE	0	0	1	Frontal gyrus
16	W	Surgery	ERTC	RT	AWD	ONB	3	4a	R0	1	1	1	1	0	0	0	6	5 frontal gyrus, 1 falx cerebri
17	W	None	ERTC	RT	AWD	ITAC	2	4b	R0	1	1	1	0	0	0	Brain	2	1 frontal gyrus, 1 falx cerebri

M: man, W: woman, CHT: chemotherapy, RT: radiation therapy, ER: endoscopic resection, ERTC: endoscopic resection with transnasal craniectomy, CER: cranioendoscopic resection, AWD: alive with disease, DOD: dead of disease, DOC: dead of other causes, ITAC: intestinal-type adenocarcinoma, SNUC: sinonasal undifferentiated carcinoma, CaNOS: carcinoma not otherwise specified, ONB: olfactory neuroblastoma, NA: not available, R0: negative margins, R1: microscopic positive margins, NE: naso-ethmoid, ACF: anterior cranial fossa, MCF: middle cranial fossa.

## Data Availability

Upon reasonable request.
